# Low Pepsinogen I/II Ratio and High Gastrin-17 Levels Typify Chronic Atrophic Autoimmune Gastritis Patients With Gastric Neuroendocrine Tumors

**DOI:** 10.14309/ctg.0000000000000238

**Published:** 2020-09-15

**Authors:** Raffaella Magris, Valli De Re, Stefania Maiero, Mara Fornasarig, Giovanni Guarnieri, Laura Caggiari, Cinzia Mazzon, Giorgio Zanette, Agostino Steffan, Vincenzo Canzonieri, Renato Cannizzaro

**Affiliations:** 1 Oncological Gastroenterology, Centro di Riferimento Oncologico di Aviano (CRO) IRCCS, Aviano, Italy;; 2 Immunopathology and Cancer Biomarkers, Centro di Riferimento Oncologico di Aviano (CRO) IRCCS, Aviano, Italy;; 3 Division of Endocrinology, Pordenone Hospital, Pordenone, Italy;; 4 Division of Diabetology, Pordenone Hospital, Pordenone, Italy;; 5 Pathology Unit, Centro di Riferimento Oncologico di Aviano (CRO) IRCCS, Aviano, Italy;; 6 DSM-Department of Medical, Surgical and Health Sciences, University of Trieste, Trieste, Italy.

## Abstract

Chronic atrophic autoimmune gastritis (CAAG) can lead to the development of gastric neuroendocrine tumors (gNETs) and can be accompanied by other autoimmune diseases. This study aimed to determine, in CAAG patients, the association of gNET development, the prevalence of autoimmune diseases other than CAAG, the association of autoimmunity, and gNET development with pepsinogen I, II, gastrin-17, and *Helicobacter pylori* infection analysis.

**METHODS:**

We determined the prevalence of gNETs and other autoimmune diseases and analyzed pepsinogen I and II, gastrin-17 serum levels, and *H. pylori* infection in all patients diagnosed with CAAG at our hospital between 2013 and 2017.

**RESULTS:**

A total of 156 patients were studied and in 15.4% was observed concomitant gNET. Approximately 68.6% had at least 1 other autoimmune disease at diagnosis of CAAG. Approximately 60.9% had autoimmune thyroiditis, followed by diabetes (19.9%) and autoimmune polyendocrine syndrome (12.8%). CAAG patients with and without gNET had similar rates of comorbidity with other autoimmune diseases, but the pepsinogen I/II ratio was lower in patients with gNET (1.6 vs 4.5, *P* = 0.018). Receiver operating characteristic curve analyses identified a pepsinogen I/II ratio <2.3 and gastrin-17 levels >29.6 pmol/L as cutoffs distinguishing CAAG patients with gNET from those without. The combined use of these cutoff correctly identified 16 of the 18 CAAG patients with gNET (*P* = 0.007). *H. pylori* infection was observed in 28.7% of cases tested but did not associate with gNET.

**DISCUSSION:**

This study suggests that a low pepsinogen I/II ratio and high gastrin-17 levels characterize patients with CAAG and gNET and confirms the frequent coexistence of CAAG with other autoimmune diseases.

## INTRODUCTION

Chronic atrophic autoimmune gastritis (CAAG) is a chronic inflammatory disease of the stomach in which an immune response is directed against the parietal cells (1) in the glands of the corpus and fundus (2). Immune destruction of the oxyntic glands leads to atrophy of the body, hypochlorhydria or achlorhydria, secondary hypergastrinemia, antral G-cell hyperplasia, and low serum levels of pepsinogen I (PGI) (3).

In CAAG, gastric mucosal atrophy can lead to the development of gastric neuroendocrine tumors (gNETs) and gastric adenocarcinoma. NETs are classified into 3 types, type I to III, according to their location, association with syndrome, clinical behavior, and the patient's clinical characteristics (4). In addition, they are graded as G1 (low), G2 (medium), and G3 (high) (4). Type I is the most frequent type of gNET, representing 70%–80% of cases (5,6), and it is associated with CAAG. Hypergastrinemia has a role in sustaining the hyperplasia of the parietal cells and thus may favor the development of gNETs (7,8). Given that a fraction of CAAG patients develop type I NETs, gastrin and other growth factors are necessary for gNET development (6). Since gNETs are largely asymptomatic, they are generally found incidentally during endoscopy. Early diagnosed gNETs have better prognosis than those diagnosed late (9). The 5-year survival of patients with gNETs ranges from 75% to 100%, and tumor stage is an independent predictor of survival (9). CAAG is often accompanied by other autoimmune diseases (10–12), including autoimmune polyglandular syndrome and nonglandular diseases such as celiac disease and rheumatoid arthritis (13).

Serological assessment of PGI, pepsinogen II (PGII), gastrin-17 (G-17), and *H. pylori* antibodies has been proposed as a noninvasive method to grade gastric atrophy (14). Low levels of PGI and a low PGI/PGII ratio are associated with severe gastric atrophy (15). PGI is produced by cells of the fundus, the acid-secreting section of the stomach, while PGII is produced by all cells of the stomach, including those in the antral section. gNETs develop from enterochromaffin-like cells, which are present mainly in the fundus (4). Gastrin is produced in the distal and antral sections and regulates acid production by stimulating enterochromaffin-like cells to release histamine that in turn stimulates parietal cells to produce hydrochloric acid (HCl). The progressive loss of parietal cells during chronic atrophic gastritis reduces the release of HCl. The resulting increase stomach pH (pH > 4) leads to an increase in gastrin production that stimulates the production of acid.

High levels of G-17 and low levels of PGI have been used to discriminate between CAAG patients and first-degree relatives of patients with gastric cancer who are at risk of gastric cancer (14,16). We previously found an association between high PGII levels and a histological diagnosis of *H. pylori* infection (14). Based on this knowledge, we hypothesize that a combined analysis of G-17 and the PGI/PGII ratio is more informative for a diagnosis of gNET than either G-17 or PGI/PGII analysis alone. Therefore, the aims of this study were (i) to determine, in a prospective cohort of patients affected by CAAG, the association of gNET development, (ii) the prevalence of autoimmune diseases other than CAAG, (iii) the association of autoimmunity, and (iv) the development of gNETs with the analysis of PGI, PGII, G-17, and anti–*H. pylori* infection.

## PATIENTS AND METHODS

We evaluated all patients with CAAG who were seen, between 2013 and 2017, at the Oncological Gastroenterology Unit of the Centro di Riferimento Oncologico of Aviano, for the prevalence of gNETs and autoimmune disorders other than CAAG. The study protocol was approved by the Institutional Review Board of the Centro di Riferimento Oncologico in 2013 (no. 14).

The study used clinical data collected from medical charts of each patient at diagnosis only (patients had not been treated with proton pump inhibitors or other drugs). Information on age, sex, and a self-reported family history of gastric cancer was collected. We recorded diagnoses of gastric cancer, which was distinguished as early gastric cancer or gNET and of other autoimmune diseases (i.e., polyendocrine syndrome, autoimmune thyroiditis, autoimmune thrombocytopenia, celiac disease, diabetes, hemolytic anemia, psoriasis, rheumatoid arthritis, Sjögren syndrome, urticaria, and vitiligo). The location of gNETs was noted, as was the grade according to the WHO classification (17). Serum levels of PGI, PGII, and G-17 were recorded, as was a diagnosis of *H. pylori* infection.

### Routine diagnostic procedures

A diagnosis of CAAG in the gastric body had been based on a combination of (i) histologically confirmed atrophy, (ii) antiparietal cell antibodies, (iii) hypergastrinemia, and (iv) low levels of PGI. Each patient had undergone esophagogastroduodenoscopy performed with a gastroscope (Olympus), with biopsies taken in the fundus, corpus, and antrum. At least 3 biopsy samples were taken from mucosecreting sections (2 antral samples plus 1 from the *incisura angularis*) and 2 from the oxyntic mucosa. Atrophy had been diagnosed histologically according to the Sydney protocol (18). Antiparietal cell antibody levels had been estimated by indirect immunofluorescence using a kit (EURO-IMMUN, Lübeck, Germany) with a cutoff of ≥1/80. Blood samples had been collected from fasting patients and processed in the morning. Serum PGI, PGII, and G-17 had been quantified using an ELISA kit (Biohit Healthcare, Helsinki, Finland). The interval for measuring G-17 was obtained by testing 200 healthy subjects. According to the parametric method, the 95% distribution reference value range was 2.5–7.0 pmol/L.

*H. pylori* infection had been diagnosed both histologically and serologically. *H. pylori* was detected by hematoxylin–eosin and Giemsa staining of biopsy sections. Serum levels of anti–*H. pylori* immunoglobulin G were determined by ELISA (Biohit Healthcare) with a cutoff of 30 EIU/mL.

### Statistical analyses

The Fisher exact test was used for testing relationships between categorical variables. The optimal decision threshold (cutoff value) for serum PGI, PGII, G-17, and anti–*H. pylori* antibodies for NET was determined using receiver operating characteristic (ROC) curve analyses, using dichotomized clinicopathological variables. Significant differences between experimental groups at different points were determined using 1‐ or 2‐way ANOVA. Serum concentrations of PGI, PGII, G-17, and anti–*H. pylori* antibodies in different groups were tested in univariate and multivariate analyses. A significance level of *P* < 0.05 was used throughout the study.

## RESULTS

### Frequency of gNETs and autoimmune diseases in CAAG patients

The study considered the clinical situation of 156 patients with CAAG at diagnosis (Table 1). Atrophy was endoscopically and histologically confirmed in all patients. Patients had a mean age of 54 years and were predominantly women. Twenty-four cases (15.4%) presented a gNET (Table 1). The 24 gNET lesions were localized to the gastric antrum (n = 2), fundus (n = 13), or corpus (n = 9). Overall, 21 cases (87.5%) were classified as grade 1 and 3 (12.5%) as grade 2 according to the WHO grading classification (17). Two of the 24 patients with gNETs had a family history of gastric carcinoma. Autoimmune disease was less frequent in patients with CAAG and NET (45.8%, Table 2) than in CAAG without NET (72.7%); the difference in frequencies was statistically significant (χ^2^ = 4.56, *P* = 0.033). gNETs associated to CAAG were also found less frequently among patients with a concomitant thyroiditis (10/24, 41.6%) than in patients without thyroiditis (58.3%); the difference did not reach a statistical significance (*P* = 0.386) (Table 2).

**Table 1.**
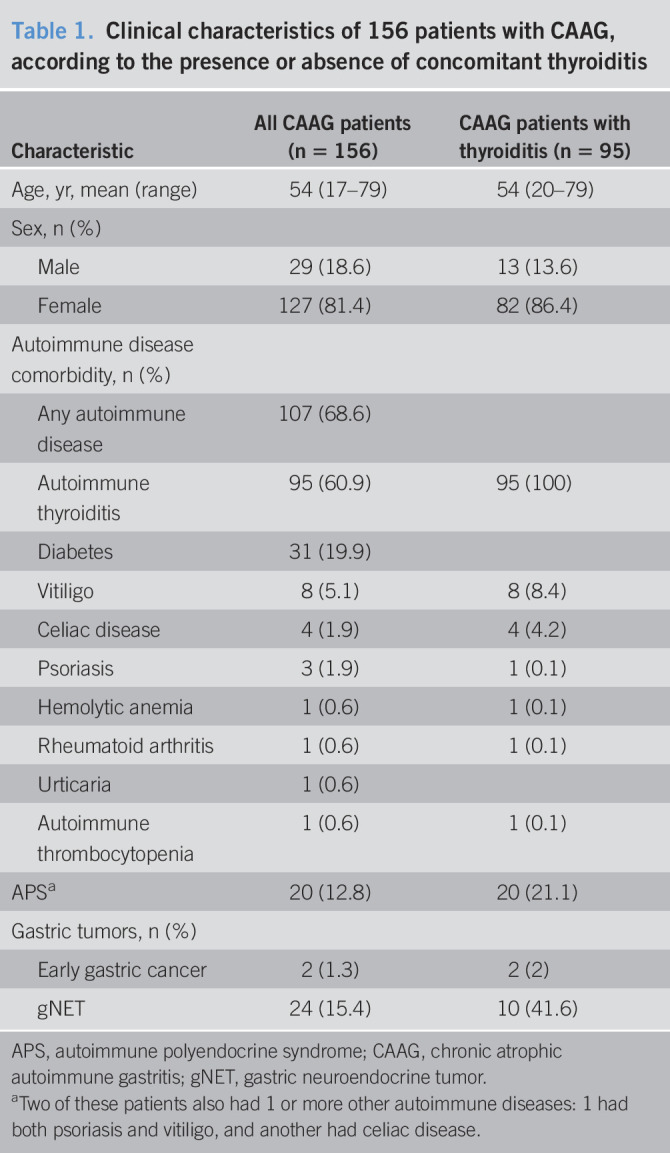
Clinical characteristics of 156 patients with CAAG, according to the presence or absence of concomitant thyroiditis

Characteristic	All CAAG patients (n = 156)	CAAG patients with thyroiditis (n = 95)
Age, yr, mean (range)	54 (17–79)	54 (20–79)
Sex, n (%)		
Male	29 (18.6)	13 (13.6)
Female	127 (81.4)	82 (86.4)
Autoimmune disease comorbidity, n (%)		
Any autoimmune disease	107 (68.6)	
Autoimmune thyroiditis	95 (60.9)	95 (100)
Diabetes	31 (19.9)	
Vitiligo	8 (5.1)	8 (8.4)
Celiac disease	4 (1.9)	4 (4.2)
Psoriasis	3 (1.9)	1 (0.1)
Hemolytic anemia	1 (0.6)	1 (0.1)
Rheumatoid arthritis	1 (0.6)	1 (0.1)
Urticaria	1 (0.6)	
Autoimmune thrombocytopenia	1 (0.6)	1 (0.1)
APS^a^	20 (12.8)	20 (21.1)
Gastric tumors, n (%)		
Early gastric cancer	2 (1.3)	2 (2)
gNET	24 (15.4)	10 (41.6)

APS, autoimmune polyendocrine syndrome; CAAG, chronic atrophic autoimmune gastritis; gNET, gastric neuroendocrine tumor.

a Two of these patients also had 1 or more other autoimmune diseases: 1 had both psoriasis and vitiligo, and another had celiac disease.

**Table 2.**
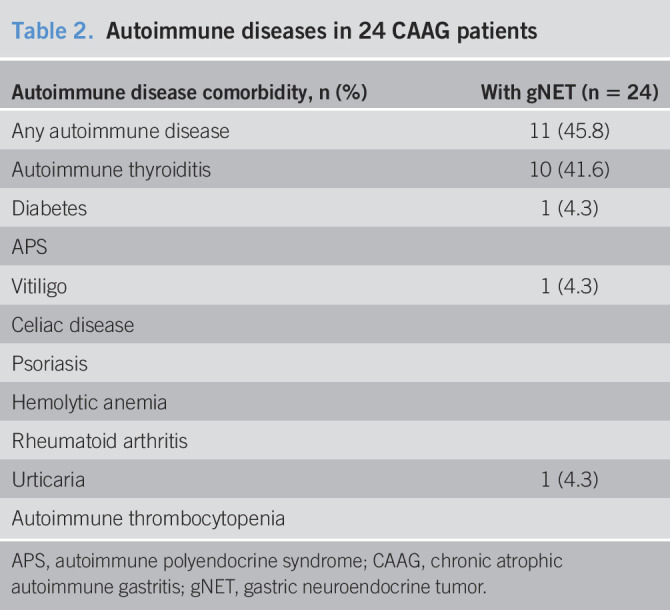
Autoimmune diseases in 24 CAAG patients

Autoimmune disease comorbidity, n (%)	With gNET (n = 24)
Any autoimmune disease	11 (45.8)
Autoimmune thyroiditis	10 (41.6)
Diabetes	1 (4.3)
APS	
Vitiligo	1 (4.3)
Celiac disease	
Psoriasis	
Hemolytic anemia	
Rheumatoid arthritis	
Urticaria	1 (4.3)
Autoimmune thrombocytopenia	

APS, autoimmune polyendocrine syndrome; CAAG, chronic atrophic autoimmune gastritis; gNET, gastric neuroendocrine tumor.

A diagnosis of at least 1 other autoimmune disorder was recorded in 107 patients (68.6%). The most prevalent comorbidity was autoimmune thyroiditis, affecting 95 cases (13.6% male, mean age of 54 years; range 20–79 years), followed by diabetes (31 cases; 36.6% male, mean age of 53 years; range 33–75 years) and autoimmune polyendocrine syndrome (APS) (20 cases; 25% male, mean age of 53 years; range 33–75 years). Other autoimmune diseases were less than 10%, as reported in Table 1.

In the same series of 156 CAAG, 2 patients (8%) developed an early gastric cancer (Table 1), the first occurred in a patient with concomitant an autoimmune thyroiditis and diabetes and the other in a patient with a thyroiditis only.

### Differences in pepsinogen and gastrin levels according to comorbidity

Data on serum PGI, PGII, and G-17 were available for 108 CAAG patients (69% of the initial group) (Table 3). Difference of serum PGI, PGII, and G-17 was found in patients with gNETs who showed a lower PGI/PGII ratio than patients without NET (1.6 vs 4.5 *P* = 0.018), while among others, patient's group resulted not statistically significant.

**Table 3.**
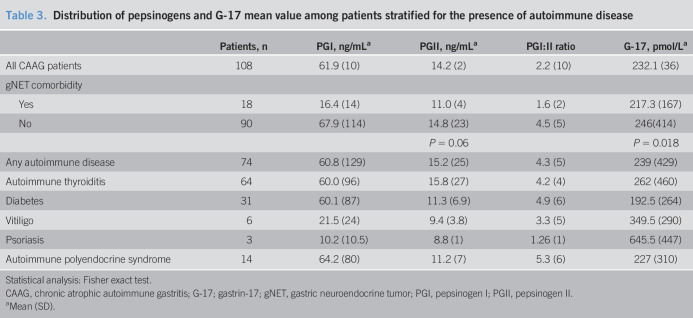
Distribution of pepsinogens and G-17 mean value among patients stratified for the presence of autoimmune disease

	Patients, n	PGI, ng/mL^a^	PGII, ng/mL^a^	PGI:II ratio	G-17, pmol/L^a^
All CAAG patients	108	61.9 (10)	14.2 (2)	2.2 (10)	232.1 (36)
gNET comorbidity					
Yes	18	16.4 (14)	11.0 (4)	1.6 (2)	217.3 (167)
No	90	67.9 (114)	14.8 (23)	4.5 (5)	246(414)
			*P* = 0.06		*P* = 0.018
Any autoimmune disease	74	60.8 (129)	15.2 (25)	4.3 (5)	239 (429)
Autoimmune thyroiditis	64	60.0 (96)	15.8 (27)	4.2 (4)	262 (460)
Diabetes	31	60.1 (87)	11.3 (6.9)	4.9 (6)	192.5 (264)
Vitiligo	6	21.5 (24)	9.4 (3.8)	3.3 (5)	349.5 (290)
Psoriasis	3	10.2 (10.5)	8.8 (1)	1.26 (1)	645.5 (447)
Autoimmune polyendocrine syndrome	14	64.2 (80)	11.2 (7)	5.3 (6)	227 (310)

Statistical analysis: Fisher exact test.

CAAG, chronic atrophic autoimmune gastritis; G-17; gastrin-17; gNET, gastric neuroendocrine tumor; PGI, pepsinogen I; PGII, pepsinogen II.

a Mean (SD).

**Figure 1.**
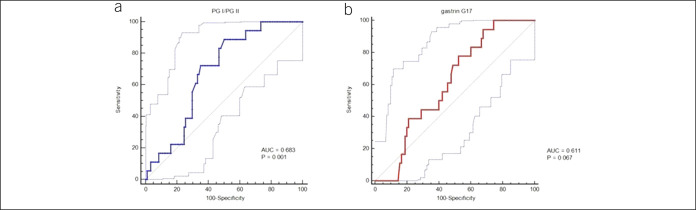
Receiver operating characteristic (ROC) curves for serum pepsinogen (PG) I/II ratio (a) and gastrin-17 (b) discriminating chronic atrophic autoimmune gastritis patients with and without gastric neuroendocrine tumors (gNETs). AUC, area under the ROC curve.

By ROC curve analysis, a PGI/PGII ratio <2.3 discriminated CAAG patients with gNET (16 of 18, 89%) from those without (31 of 90, 34%), with an area under the curve of 0.683 (95% confidence interval, 0.576–0.761, *P* = 0.001) (Figure 1). No difference was observed between CAAG patients with and without gNET by using the G-17 parameter (Figure 1).

**Figure 2.**
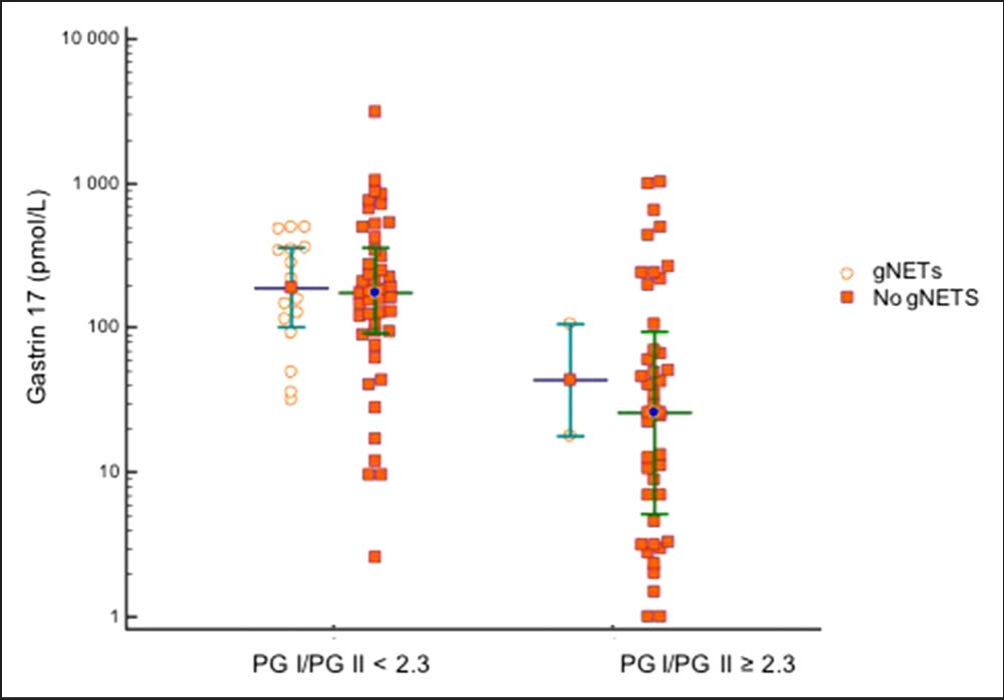
Gastrin-17 levels among chronic atrophic autoimmune gastritis patients, by pepsinogen (PG) I/II ratio and presence or absence of gastric neuroendocrine tumor (gNET).

Patients with a PGI/PGII ratio ≥2.3 had lower G-17 levels than those with a ratio <2.3, although the difference did not reach a statistical significance (Figure 2). A cutoff for G-17, determined by ROC analysis, was >29.6 pmol/L. Combining both criteria discriminated CAAG patients with gNETs from those without gNET. Specifically, a PGI/PGII ratio <2.3 and G-17 >29.6 pmol/L identified 16 (88.9%) of 18 patients with gNET and 44 (48.8%) of 90 patients without gNET (Fisher exact test, *P* = 0.007).

### *Helicobacter pylori* infection

*H. pylori* infection was available in 136 CAAG cases (87.1%), and 39 (28.7%) patients were positive. Among these, 3/20 (15%) had gNETs, 23/86 (26.7%) had autoimmune thyroiditis, 5/23 (21.7%) had diabetes, and 2/16 (12.5%) had APS (Table 4).

**Table 4.**
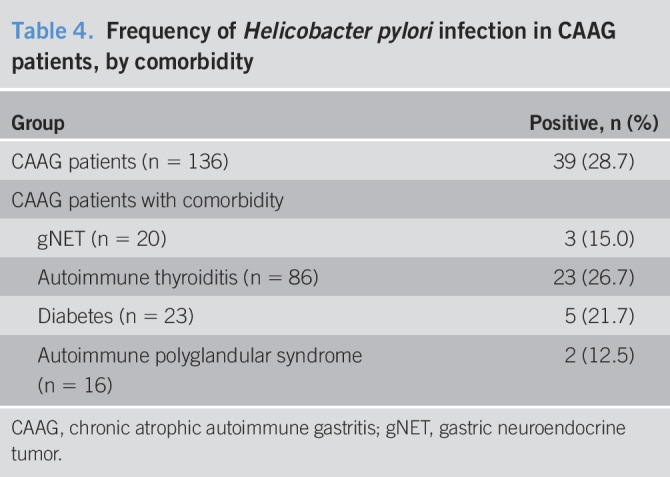
Frequency of *Helicobacter pylori* infection in CAAG patients, by comorbidity

Group	Positive, n (%)
CAAG patients (n = 136)	39 (28.7)
CAAG patients with comorbidity	
gNET (n = 20)	3 (15.0)
Autoimmune thyroiditis (n = 86)	23 (26.7)
Diabetes (n = 23)	5 (21.7)
Autoimmune polyglandular syndrome (n = 16)	2 (12.5)

CAAG, chronic atrophic autoimmune gastritis; gNET, gastric neuroendocrine tumor.

Results of PGII analysis showed that the mean of PGII levels was slightly higher in patients with *H. pylori* infection, although the difference did not reach a statistical difference and it is independent of the presence of gNET (Table 5).

**Table 5.**
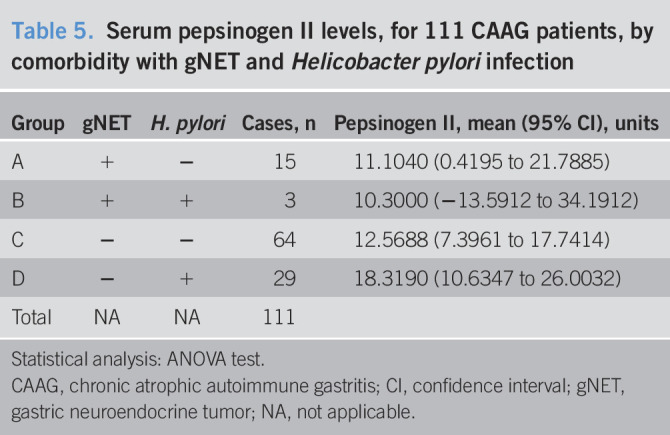
Serum pepsinogen II levels, for 111 CAAG patients, by comorbidity with gNET and *Helicobacter pylori* infection

Group	gNET	*H. pylori*	Cases, n	Pepsinogen II, mean (95% CI), units
A	+	−	15	11.1040 (0.4195 to 21.7885)
B	+	+	3	10.3000 (−13.5912 to 34.1912)
C	−	−	64	12.5688 (7.3961 to 17.7414)
D	−	+	29	18.3190 (10.6347 to 26.0032)
Total	NA	NA	111	

Statistical analysis: ANOVA test.

CAAG, chronic atrophic autoimmune gastritis; CI, confidence interval; gNET, gastric neuroendocrine tumor; NA, not applicable.

## DISCUSSION

CAAG is often associated with the development of gNETs and APSs (19), but the incidence of gNETs and specific autoimmune diseases in CAAG is not known.

This study by investigating correlation between CAAG, gNETs, and coexisting autoimmune diseases shows a strong association of CAAG with development of gNETs (15.4%) and confirms (8,20–24) the frequent presence of other autoimmune diseases (68.6%) in particularly with autoimmune thyroiditis (60.9%) and diabetes (19.9%) in CAAG patients.

The prevalence of gNETs we found (15.4%) is high compared with previous studies: Vannella et al. (25) reported 2.4% of coexisting CAAG and gNETs at the diagnosis (24), and other studies reported rates between 1% and 12.5% (23–27). As mentioned above, NETs are classified into 3 types (type I to III) and graded as G1, G2, and G3 based on prognostic parameters of the proliferative index Ki-67, the mitotic count (number of mitosis × high power field [HPF]: mm^2^ area within the tumor) (4) G1: Ki-67 ≤2%, <2 mitoses/10 HPF; G2: K-67 3%–20%, 2–20 mitoses/10 HPF, G3: Ki-67 >20%, >20 mitoses/10 HPF (17). Early diagnosis of a gNET is important to improve the prognosis. Simple surveillance or local endoscopic resection is recommended in tumors smaller than 20 mm in size and without features of invasion of muscolaris propria or metastasis, regardless the tumor number. Surgical resection or endoscopic resection is recommended for tumors that are greater than 20 mm in size, whether single or multiple (5). In particular, endoscopic submucosal dissection is useful and guarantees a superior complete resection compared with endoscopic mucosal resection for removal of submucosal tumors (5).

Patients with CAAG have autoantibodies against parietal cells of the gastric mucosa arising from a genetic autoimmune process or an autoimmune process due to a hypothesized mimicry between epitopes of *H. pylori* antigens and parietal cell antigens in susceptible individuals. Atrophy caused by antiparietal cell antibodies, specifically in the corpus, reduces the production of PGI and therefore the secretion of G-17 in an effort to stimulate parietal cells to produce HCl and re-established the normal gastric pH. Serum PGI, PGII, and G-17 are indicators of morphologic and functional changes in cells of the stomach and are used to identify and grade atrophy (14,28–30).

In our series, patients with concomitant CAAG and gNETs were more likely to have a PGI/PGII ratio <2.3 and G-17 ≥29.6 pmol/L than patients without gNETs (*P* = 0.007), suggesting, for the first time, that it is advantageous to test the combination of the 2 variables than any single variable. Together, these criteria well characterized CAAG patients with gNET, since among 89 individuals with CAAG without gNET, the combined PGI/PGII <2.3 and G-17 ≥29.6 pmol/L criteria were met only in 2 cases (2.2%, *P* < 0.001).

The pathologic cause of CAAG is primarily a genetic autoimmune disorder, but environmental causes, such as *H. pylori* infection, may favor the development of autoimmunity. Although it is known that PGII levels correlate with *H. pylori* infection (14,31), in our series, the mean PGII level was slightly higher in patients without gNET, but this difference did not reach statistical significance. This finding suggests that gNET is unrelated to *H. pylori* infection, as a previous study reported (32).

In conclusion, we focused our attention on the high prevalence of gNETs in our series and we identified different coexisting autoimmune diseases in patients with CAAG.

We found that a low PGI/PGII ratio (<2.3) in concomitance with a G-17 higher than 29.6 pmol/L characterizes patients with CAAG and gNET. This finding suggests the existence of 2 CAAG subgroups. The reasons for this difference require further studies.

## CONFLICTS OF INTEREST

**Guarantor of the article:** Renato Cannizzaro, MD.

**Specific author contributions:** R.M.: designed the study, developed the methodology, acquired data, and wrote and reviewed the manuscript; she approved the final draft submitted. V.D.R.: designed the study, developed the methodology, acquired data, and wrote and reviewed the manuscript; she approved the final draft submitted. S.M.: identified and recruited patients; she approved the final draft submitted. M.F.: identified and recruited patients; she approved the final draft submitted. G.G.: identified and recruited patients; the author approved the final draft submitted. L.C.: reviewed the manuscript; she approved the final draft submitted. C.M.: identified and recruited patients; she approved the final draft submitted. G.Z.: identified and recruited patients; he approved the final draft submitted. A.S.: performed laboratory analyses; he approved the final draft submitted. V.C.: performed histological analyses; he approved the final draft submitted. R.C.: conceived, designed, and supervised the study, identified and recruited patients, wrote the manuscript, and critically reviewed the manuscript; he approved the final draft submitted.

**Financial support:** The project was supported by the Italian Ministry of Health (Ricerca Corrente) [no grant number provided]. CRO 5X1000_2010_MdS, Italy.

**Potential competing interests:** None to report.

**Study Highlights**

**WHAT IS KNOWN**

✓ Patients with CAAG often develop gNETs and have other autoimmune diseases.
✓ PGI and PGII and G-17 are important in predicting gastric mucosa atrophy.
✓ PGII levels may correlate with *H. pylori* infection.

**WHAT IS NEW HERE**

✓ Patients with both CAAG and gNETs have a lower PGI/II ratio and higher G-17 levels than CAAG patients without such tumors.
✓ Combined application of <2.3 pepsinogen ratio and >29.6 pmol/L G-17 correctly identified CAAG patients with gNETs.
✓ *H. pylori* infection did not associate with the prevalence of gastric neuroendocrine tumors in CAAG patients.

**TRANSLATIONAL IMPACT**

✓ Determination of PGI and PGII and G-17 levels in CAAG patients may help identify those at risk of gastric neuroendocrine tumors.
